# The importance of early intraoperative ultrasonography and the relationship between surgical technique and allograft function in patients with compartment syndrome following renal transplantation surgery

**DOI:** 10.55730/1300-0144.6180

**Published:** 2026-01-25

**Authors:** Burak YAĞDIRAN, Emre KARAKAYA, Ravana AHMADLI, Adem ŞAFAK, Yağmur KURTULUŞ, Nedim ÇEKMEN, Meriç YAVUZ ÇOLAK, Mehmet HABERAL

**Affiliations:** 1 Department of Radiology, Faculty of Medicine, Başkent University, Ankara, Turkiye; 2 Department of General Surgery, Faculty of Medicine, Başkent University, Ankara, Turkiye; 3 Başkent University, Faculty of Medicine, Ankara, Turkiye; 4 Department of Anesthesiology and Intensive Care Unit, Faculty of Medicine, Başkent University, Ankara, Turkiye; 5 Department of Biostatistics, Başkent University, Ankara, Turkiye

**Keywords:** Renal transplantation, renal allograft compartment syndrome, Doppler ultrasonography, resistive index, peak systolic velocity

## Abstract

**Background/aim:**

In rare cases, patients undergoing renal transplantation **(**RT) may develop renal allograft compartment syndrome (RACS) if the renal volume is larger than the retroperitoneal space prepared in the iliac fossa. In such cases, the optimal treatment is early decompression surgery. The primary aim of the present descriptive and comparative study is not to identify the optimal surgical technique, but rather to evaluate the role of early Doppler ultrasonography (DUSG) in the recognition of renal allograft compartment syndrome and to assess outcomes following timely decompressive interventions.

**Materials and methods:**

A total of 275 patients were examined retrospectively for the study, including 247 with muscle and fascia closure and 28 with skin-only closure.

**Results:**

Only 28 (10.18%) of the patients with RACS underwent permanent fasciotomy and skin-only closure. No statistically significant difference was observed in the mean urine output between the two groups on any postoperative day, assessed based on the renal artery resistive index and peak systolic velocity.

**Conclusion:**

RACS should be considered in patients with large-sized allografts and small parailiac retroperitoneal spaces to address the inherent anatomical restrictions. Although early diagnosis with DUSG is essential for evaluating graft dysfunction in these patients, early decompression surgery should be considered crucial for the preservation of graft function in those with suspected RACS.

## 1. Introduction

Renal transplantation (RT) is an essential treatment modality in patients with end-stage renal failure given its benefits both to survivability and quality of life [1]. In rare cases, patients undergoing RT can develop renal allograft compartment syndrome (RACS) if the renal volume exceeds the available retroperitoneal space in the iliac fossa and if the kidney vasculature, ureter, and abdominal muscles are shorter than usual [2]. A review of the literature uncovered only a few case reports and small case series on RACS [3]. Graft perfusion is impaired by compression of the renal parenchyma or vascular structures [4]. In such situations, renal artery flow is initially good, but venous flow becomes impaired as a result of abdominal wall pressure on the renal vein, and subsequently renal venous pressure increases, the graft vessels become occluded, and urine outflow decreases.

Intraoperative DUSG should be used to guide graft placement for diagnostic purposes, and urine output should be monitored in the early postoperative period in cases with normal DUSG findings. If urine volume decreases during follow-up, DUSG should be repeated without delay to identify RACS. Decreased venous flow, increased resistance in the renal artery, parvus tardus flow patterns in the distal branches, or reverse diastolic flow in the artery on DUSG are suggestive of RACS, and emergency decompression surgery is advised. RACS is a preventable cause of allograft dysfunction with early surgical intervention. In such cases, graft dysfunction can be prevented early by decompression surgery. Diagnosis and treatment are confirmed when the edematous appearance of the graft decreases and graft vascularity normalizes on DUSG [4,5].

The primary aim of the present study is to emphasise the importance of routine intraoperative and early postoperative DUSG for the diagnosis of RACS. As a secondary aim, the graft function and the likelihood of lymphocele development in the postoperative period are compared between patients with suspected RACS who underwent decompression surgery and those without suspected RACS.

## 2. Materials and methods

**Figure 1:**
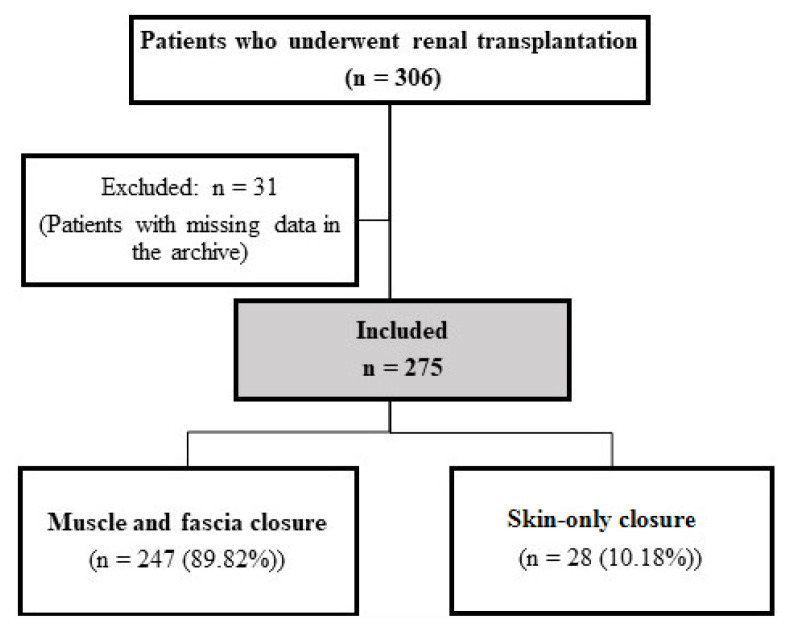
Study flow chart.

Approval for this retrospective study was granted by the Ethics Committee of our university (KA25/107), after which, the records of 306 patients who underwent RT surgery in our hospital for the treatment of compartment syndrome between 2018 and 2023 were reviewed. Patients underwent either muscle and fascia closure or skin-only fascial mesh closure procedures. Subsequently, 31 patients were excluded from the study due to incomplete DUSG and laboratory data, and missing 7-day urine output and lymphocele drain volume records in the archive. The study was thus completed with 275 patients of whom 247 underwent muscle and fascia closure procedures and 28 with skin-only closure (Figure 1).

The surgical closure technique was determined based on intraoperative or early postoperative clinical assessment rather than by random allocation, resulting in unequal and clinically heterogeneous groups. Patients undergoing skin-only closure procedures thus represent a clinically distinct, higher-risk subgroup in which treatment selection was based on clinical necessity rather than a predefined allocation. The retrospective nature of the study and the mismatch in group sizes precluded the performance of a formal power analysis prior to the study. The relatively small number of patients in the skin-only closure group may have reduced the statistical power and increased the risk of Type II error in the study, meaning that the statistical analyses are primarily descriptive and exploratory.

The resistive index (RI) and peak systolic velocity (PSV) determined during DUSG follow-up were used to evaluate postoperative graft function in the patients. Creatinine decline rates, total urine volumes in the first 7 days, and outputs of lymphocele drains placed on postoperative day 1 were obtained from archival records, along with records of patients requiring reinsertion of lymphocele drains.

Renal artery RI and PSV values at postoperative days 3 and 7, and months 1, 3, and 6 were used to determine graft function. Creatinine values on day 7, and months 1 and 3 were used to determine the rate of creatinine decline. Urine volumes were compared based on the total urine output up to postoperative day 7. In addition to evaluating graft function, we compared the total fluid outputs from the lymphocele drain catheter.

The primary aim of this descriptive and comparative study is not to identify the optimal surgical technique, but to evaluate the role of early DUSG in the recognition of RACS and to assess outcomes following timely decompressive interventions. The RI and PSV values on DUSG, creatinine decline rate, urine volume, lymphocele drain fluid outputs, and drainage times recorded for both surgical techniques were analysed. For this reason, any analysis of the surgical closure techniques should be regarded as descriptive and exploratory rather than confirmatory.

### 2.1. Statistical analysis

Statistical analyses were performed using IBM SPSS 25.0 software (IBM Corporation, Armonk, NY, USA). The normality of the variables was assessed using the Kolmogorov–Smirnov test. Descriptive data were presented as mean ± standard deviation and median (min–max) values, and categorical variables as frequencies and percentages. Relationships between categorical variables were examined with Pearson’s chi–square Test when the assumptions were met, and with the Freeman–Halton Test (Fisher Exact Test) when they were not. Differences in quantitative variables between two independent groups were determined using the Wilcoxon rank-sum test when parametric assumptions were not met. p values of <0.05 were considered statistically significant.

## 3. Results

Graft-related outcomes were descriptively evaluated according to the surgical closure approach as an exploratory analysis. Only 28 patients (10.18%) with RACS underwent permanent fasciotomy and skin-only closure procedures. The primary factor used for the evaluation of graft function was the rate of decline in creatinine values during follow-up. The creatinine values of the groups on postoperative day 7, and months 1 and 3 were compared. The absence of statistical significance should not be interpreted as evidence of no clinical difference, particularly in underpowered comparisons. The between-group comparisons yielded nonsignificant results and consistently small effect sizes across all evaluated outcomes. No statistically significant differences were observed between the groups with respect to serum creatinine levels (p = 0.945; p = 0.587; p = 0.475) (Table 1).

**Figure 2:**
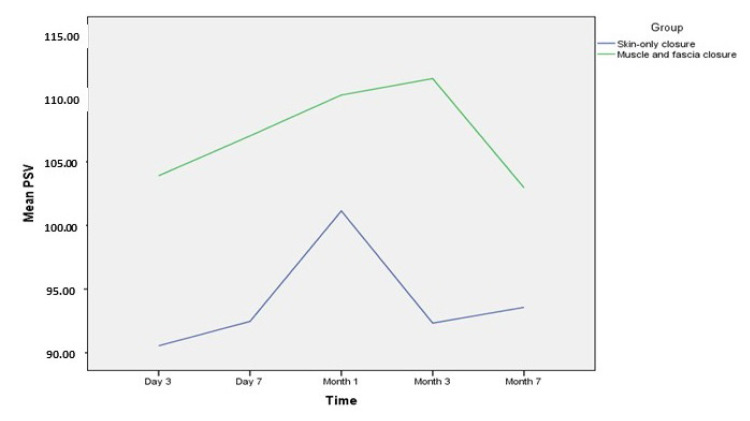
Time dependent changes of PSV values according to surgical technique.

**Figure 3:**
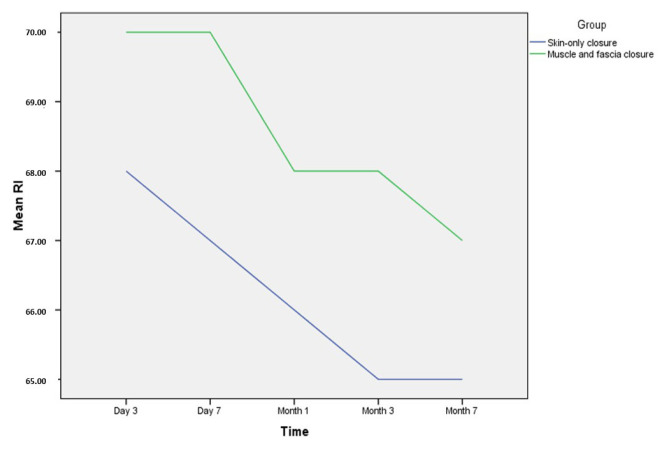
Time dependent changes in resistive index (RI) according to surgical technique.

Graft function was further evaluated based on total urine output in the first 7 days. The mean urine production was 4600.00 ± 1913.06 mL in the open fascia group and 4616.23 ± 1805.27 mL in the closed muscle and fascia group. No statistically significant differences were observed between the groups with respect to the total amount of urine in the first 7 days (p = 0.732) (Table 2). Graft function was also compared based on an evaluation of renal artery PSV and RI values at postoperative days 3 and 7, and months 1, 3, and 6. No statistically significant differences were observed between the groups at any of the postoperative timepoints (Table 3). The time-dependent variations in PSV and RI values according to surgical technique are presented in Figures 2 and 3, respectively.

Aside from graft function, total drainage volume was also assessed based on total lymphocele catheter output, and the number of patients requiring redrainage after catheter removal was recorded. No statistically significant differences were observed between the groups with respect to the lymphocele volumes identified in the parailiac region associated with the two surgical procedures (Tables 4 and 5). The mean total lymphocele volume was 1897.40 ± 2846.18 mL in the skin-only closure group and 991.00 ± 1337.42 mL in the muscle and fascia closure group, and the difference was not statistically significant (p = 0.446) (Table 4). Redrainage was required in five patients (17.869%) in the skin-only closure group and 15 patients (6.07%) in the muscle and fascia closure group, and this difference was also not statistically significant (p = 0.091) (Table 5).

## 4. Discussion

It is plausible that the early diagnosis and timely decompression in the skin-only closure group attenuated differences that might otherwise have emerged in renal function or Doppler parameters. For this reason, the findings primarily reflect the benefits of early intervention rather than providing a definitive comparison of the two closure techniques.

RACS is a poorly understood cause of early graft dysfunction that can be treated with early intervention and prevented by careful examination of the graft and its position in the retroperitoneal space opened for grafting [5]. A review of the literature revealed few studies of RACS to date, and a reported incidence of approximately 2% [6]. The 10% RACS rate observed in the present study is thus higher than that reported in previous studies. The case report published by El-Bandar et al. suggests that this incidence rate may be underestimated due to the lack of recognition of RACS, leading graft dysfunction to be wrongly attributed to such conditions as thrombosis or delayed graft function. The same study claims that transplanted renal vein thrombosis may have occurred in some cases due to RACS [7]. Similarly, recently published studies by Otludil et al., Bamaniya et al., and Bañuelos et al. all report significant deficiencies in the literature related to RACS diagnoses, claiming that the true incidence rates may be higher than those reported in earlier studies [5,8,9]. We believe the high incidence rate reported in the present study may be attributable to the close intraoperative and postoperative monitoring with DUSG, the detailed clinical assessment of RACS, and the early recognition of the condition.

In two animal studies conducted by Doty et al., renal vein compression alone was noted to decrease renal blood flow and glomerular filtration, increase plasma renin activity, and increase urinary protein leakage in abdominal compression syndrome [10]. Such changes can be wholly or partially reversed by decreasing renal venous pressure, as in cases with abdominal decompression in acute abdominal compartment syndrome. Both renal vein and parenchymal compression are significant etiologic factors in RACS. Decompression surgery is vital for the preservation of graft function in cases in which DUSG findings are indicative of RACS.

Ortiz et al. assessed the risk of RACS based on such parameters as the length and width of the allograft, the weight of the recipient, the body mass index of the donor, the incision site, and previous peritoneal dialysis history, and reported a correlation only between graft length and the risk of RACS [3]. In all seven RACS cases diagnosed in the study, graft function was preserved with early DUSG diagnosis and prompt decompression surgery, similar to the present study [3].

Koss et al. described a case in which a kidney previously transplanted to the iliac fossa was placed intraperitoneally after excessive intra-abdominal distension led to the development of compartment syndrome [11]. Ball et al. reported the intraperitoneal placement of transplanted kidneys for the treatment of RACS in eight patients [6]. Decompression surgery was sufficient in all patients in the present study, and none required intraperitoneal transplantation.

In the present study, DUSG data, creatinine decline rates, and total urine outputs over the first 7 postoperative days were compared for the evaluation of graft function in patients who underwent reexploration for RACS in the postoperative period and in those who did not develop RACS and underwent routine transplantation surgery. No significant differences were observed between the groups in any of the mentioned parameters, suggesting that the early diagnosis and decompression with reexploration contributed to the preservation of graft function.

Aside from our evaluation of graft function, we also conducted an analysis of the number of lymphoceles developing in the para-iliac area to identify any differences in the muscle and fascia and skin-only closure groups. To this end, we examined the total drainage volume based on the total lymphocele catheter output and the need for redrainage after catheter removal; however, our statistical analysis revealed no significant difference between the two groups in terms of the number of lymphoceles. Redrainage was required in five cases (17.869%) in the skin-only closure and 15 cases (6.07%) in the muscle and fascia closure groups, and the difference was not significant.

The marked disparity in patient distribution between the two surgical groups represents a significant limitation of this study, although the most important limitation was its retrospective design. All data on urine volume, drainage, and lymphocele evaluation were obtained from patient records in the archive, and we accepted the values we used as optimal. DUSG data were obtained from images in the image archive, and were considered to be more objective than the other data. The disparity in the sample sizes of the groups may have reduced the statistical power of the study and prevented the detection of significant differences, particularly for less frequently occurring outcomes. However, this distribution reflects the situation in real-world clinical practice, in which the second technique is applied selectively. The findings of the study should thus be interpreted cautiously and validated in larger, prospectively balanced cohorts. Although group imbalance remains an inherent limitation of the study, the finding that effect sizes were uniformly small across all outcomes and time points suggests that significant clinically meaningful differences were unlikely to have been overlooked. It should be kept in mind, however, that small effect sizes should not be considered evidence of clinical equivalence, particularly in a nonrandomised and potentially underpowered setting.

One important consideration that should be noted when interpreting the results is the potential for selection bias and clinical heterogeneity across the study groups. The patients who underwent skin-only closure procedures were identified based on intraoperative or early postoperative suspicion of RACS, and so differed systematically from those undergoing standard muscle and fascia closure. As a result, the findings should not be interpreted as a direct comparison of closure techniques in comparable patient populations, as the primary aim of the study is to assess the outcome following the early recognition of suspected RACS and timely decompressive interventions, which may have contributed to the preservation of graft function.

The absence of statistically significant differences across multiple outcomes should also be interpreted with caution. Statistical nonsignificance does not imply clinical equivalence, particularly when the statistical power of the study is limited and the group imbalance is marked. Therefore, the current findings prevent definitive conclusions being drawn regarding the comparative clinical effectiveness of the two surgical approaches.

It is also possible that early diagnoses using DUSG and timely decompressive interventions contributed to the preservation of graft function, thereby mitigating differences that might otherwise have been observed. In this context, the results may reflect the effectiveness of early recognition and intervention rather than an inherent similarity between surgical techniques.

The findings of the present study clarify the importance of early diagnosis and timely decompression in patients with suspected RACS. The role of DUSG in the early recognition of the condition appears central to clinical decision-making and may contribute to the preservation of graft function. In contrast, analyses examining the association between surgical closure techniques and graft-related outcomes should be interpreted as exploratory. Given the selection bias, clinical heterogeneity, and limited statistical power of the present study, these analyses should be considered hypothesis-generating, and do not permit definitive conclusions to be drawn regarding the comparative effectiveness of closure techniques.

In this retrospective analysis, RACS was observed in a limited number of patients undergoing RT. While no statistically significant differences were observed between the evaluated groups, these findings should be interpreted with caution, given the substantial imbalance in group sizes and the limited statistical power of the study. Rather than indicating equivalence between surgical approaches, the results highlight clinical scenarios in which a heightened awareness of RACS may be warranted. Careful preoperative assessment and allograft positioning within a multidisciplinary framework appear necessary for risk identification. Early DUSG may contribute to the timely evaluation of graft dysfunction in the intraoperative and early postoperative periods, and early decompression may be considered in patients with suspected RACS to support the preservation of graft function. As definitive conclusions regarding the comparative effectiveness of the two procedures cannot be drawn from the present data, larger, prospective, and preferably randomised studies with balanced cohorts are warranted to identify the relevant risk factors and diagnostic strategies for the optimal management of RACS.

## 5. Conclusion

The primary aim of this study has been to highlight the clinical benefits of the early recognition and decompressive management of suspected RACS. Given the nonrandomised design and inherent differences between the patient groups, the results should not be interpreted as a direct comparison of surgical closure techniques.

While no statistically significant differences were observed between the study groups, the findings should not be construed as evidence of clinical equivalence. The presented results should rather be viewed as descriptive observations influenced by early diagnosis and intervention.

RACS should be considered in patients undergoing RT, especially those with large-sized allografts and anatomically small parailiac retroperitoneal spaces. Anatomical vascular structures and allograft locations of RT patients should be analysed by a multidisciplinary team as part of the preoperative evaluation. For this reason, early DUSG is extremely important when evaluating graft dysfunction during surgery and in the early intraoperative and postoperative periods. Prompt decompression surgery in patients with suspected RACS is crucial for the preservation of graft function, although more effective, prospective, and randomised studies are needed.

## Figures and Tables

**Table 1:** Creatine values in groups.

N=275	Skin-only closure (n = 28)		Muscle and fascia closure (n = 247)		p-value
	Mean±s	Median (min–max)	Mean±s	Median (min–max)	
**Day 7**	1.78±1.28	1.57 (0.38–5.47)	1.79±1.18	1.38 (0.34–6.62)	0.945
**Month 1**	1.58±1.26	1.29 (0.50–5.35)	1.30±0.66	1.15 (0.42–5.51)	0.587
**Month 3**	1.69±1.65	1.48 (0.66–8.34)	1.34±0.54	1.27 (0.40–4.70)	0.475

* ; p < 0.05 significant; Mann**–**Whitney U test; s: standard deviation.

**Table 2:** Total urine volumes in the first 7 days between groups.

N = 275	Skin-only closure (n = 28)		Muscle and fascia closure (n = 247)		p-value
	Mean±s	Median (min–max)	Mean±s	Median (min–max)	
Total urine volume (mL)	4600.00 ±1913.06	4840.00 (20.00–6800.00)	4616.23±1805.27	4600.00 (0.00–9000.00)	0.732

* ; p < 0.05 significant; Mann**–**Whitney U test; s: standard deviation.

**Table 3:** PSV and RI values in groups.

N = 275	Skin-only closure (n = 28)		Muscle and fascia closure (n = 247)		p-value
	Mean±s	Median (min–max)	Mean± s	Median (min–max)	
Day 3 PSV	90.55±37.96	83.50 (30.00–199.00)	103.90±42.57	96.50 (37.00–270.00)	0.142
Day 3 RI	0.68±0.07	0.69 (0.48–0.82	0.70±0.09	0.69 (0.46–0.91)	0.545
Day 7 PSV	92.46±36.48	86.50 (43.00–209.00)	107.03±45.62	98.00 (29.00–305.00)	0.110
Day 7 RI	0.67±0.07	0.67 (0.56–0.83)	0.70±0.08	0.69 (0.51–0.95)	0.084
Month 1 PSV	101.14±34.10	99.00 (40.00–178.00)	110.24±39.66	105.00 (44.00–291.00)	0.392
Month 1 RI	0.66±0.05	0.66 (0.56–0.78)	0.68±0.07	0.67 (0.47–0.93)	0.129
Month 3 PSV	92.33±33.75	87.00 (48.00–164.00)	111.53±68.71	102.00 (10.00–900.00)	0.099
Month 3 RI	0.65±0.06	0.66 (0.54–0.85)	0.68±0.08	0.67 (0.38–0.93)	0.157
Month 7PSV	93.57±34.09	93.00 (54.00–185.00)	102.96±36.27	102.00 (35.00–252.00)	0.151
Month 7 RI	0.65±0.05	0.65 (0.54–0.75)	0.67±0.07	0.67 (0.51–0.96)	0.184

* ; p < 0.05 significant; Mann–Whitney U test; s: std deviation; PSV, peak systolic velocity (PSV); RI, renal artery resistive index.

## Data Availability

The datasets generated or analysed during the study are available from the corresponding author upon reasonable request.
